# Evaluation of Neuroprotective Effect of Thymoquinone Nanoformulation in the Rodent Cerebral Ischemia-Reperfusion Model

**DOI:** 10.1155/2016/2571060

**Published:** 2016-09-20

**Authors:** Xiao-Yu Xiao, Ying-Xian Zhu, Ju-Yuan Bu, Guo-Wei Li, Jian-Hui Zhou, Shao-Peng Zhou

**Affiliations:** ^1^Department of Anaesthesiology, Fifth Affiliated Hospital, Sun Yat-Sen University, Zhuhai, Guangdong 519000, China; ^2^Department of Gastrointestinal Surgery, Fifth Affiliated Hospital, Sun Yat-Sen University, Zhuhai, Guangdong 519000, China; ^3^Department of Spine Surgery, Fifth Affiliated Hospital, Sun Yat-Sen University, Zhuhai, Guangdong 519000, China; ^4^Department of Laboratory, Fifth Affiliated Hospital, Sun Yat-Sen University, Zhuhai, Guangdong 519000, China

## Abstract

The purpose of the present study was to evaluate the neuroprotective efficacy of optimized thymoquinone loaded PLGA-chitosan nanoparticles delivered via nose to brain route in the rodent cerebral ischemia-reperfusion model. The neuroprotective efficacy of the optimized thymoquinone loaded PLGA-chitosan nanoparticles was evaluated in middle cerebral artery occluded rats by various pharmacodynamic and biochemical studies. The pharmacokinetics of thymoquinone loaded PLGA-chitosan nanoparticles in the brain and blood plasma together with qualitative localization of florescent labelled PLGA-chitosan nanoparticles in brain tissues were also determined. Intranasal delivery of optimized thymoquinone loaded PLGA-chitosan nanoparticles (183.5 ± 8.2 nm, 33.63 ± 2.25 mV) to brain significantly reduced the ischemia infarct volume and enhanced the locomotor activity and grip strength in the middle cerebral artery occluded rats. Biochemical studies showed that intranasal delivery of thymoquinone loaded PLGA-chitosan nanoparticles significantly reduced the lipid peroxidation but elevated the glutathione, catalase, and superoxide dismutase in the brain of middle cerebral artery occluded rats. The pharmacokinetic and localization studies showed that thymoquinone loaded PLGA-chitosan nanoparticles facilitated the delivery of thymoquinone to brain by intranasal nose to brain transport pathways and enhanced their pharmacokinetic profile in brain tissues. Thus, intranasal delivery of thymoquinone loaded PLGA-chitosan nanoparticles to brain could be potentially used for the neuroprotection and treatment of cerebral ischemia.

## 1. Introduction

Stroke is among the most leading causes of disability and death worldwide [1]. According to published reports, 1 in 6 persons will suffer at least one stroke in their lifetime which would cause death or disability and have magnanimous financial impact on health systems worldwide [1, 2]. About 80% of these strokes are due to cerebral ischemia caused by embolic or nonembolic occlusion of a vital cerebral artery triggering the neuronal death, cerebral damage, morbidity, and mortality [2, 3]. Further, there is large clinical variability in these strokes in terms of severity of ischemia, localization, duration, and causes which continue to make it one of the most challenging CNS diseases. The ischemia-reperfusion injury in brain has been found to produce large quantities of reactive oxygen species (ROS, e.g., hydroxyl radical, superoxide, peroxynitrite, and hydrogen peroxide) leading to oxidative stress which aggravates the damage done by cerebral ischemia [2–4]. As tissues of brain are more vulnerable to oxidative damage, pharmacological intervention for oxidative damage has been found to have promising role in ischemia stroke therapy [4]. In recent years, thymoquinone (TQ) extracted from seeds of* Nigella sativa* (Ranunculaceae) has been discovered to diminish ROS-mediated reactions and save the neurons from reperfusion-induced neuronal damage in rodents [5, 6]. However, the brain delivery of these phytochemicals had been challenging as the presence of blood brain barrier (BBB) and blood-cerebrospinal fluid barrier prevents the free circulation of drug between blood and the central nervous system (CNS) [7]. Although these barriers are vital for normal physiological functions of the brain and spinal cord, they also present a significant hurdle in the treatment of various brain disorders [7]. These barriers also restrict the efficacy of different potential drug delivery systems delivered by oral, intravenous, transdermal, and other routes [7, 8]. In past few years, intranasal (i.n.) nose to brain delivery has emerged as a novel noninvasive technique for transporting therapeutic agents to the CNS [8, 9]. Nose to brain drug delivery is possible due to unique connection provided by the olfactory and/or trigeminal nerve system present between the olfactory epithelium and the CNS, bypassing the BBB [8, 9]. Recently, a large number of publications have reported the nose to brain delivery of many drugs due to its obvious benefits, for example, avoidance of BBB and hepatic first pass metabolism, noninvasiveness, and ease of administration [8–11].

The objective of the present research work was to formulate a nanosized drug delivery system of TQ for evaluation of its neuroprotective efficacy in cerebral ischemia-reperfusion in rats. An additional aim of the current study was to determine the pharmacokinetics and brain localization of nose to brain delivered thymoquinone loaded PLGA NPs (TQ-PLGA NPs). For development of drug delivery system, polymeric nanoparticles composed of poly(lactide-co-glycolide) (PLGA) and chitosan (CN) were chosen because of their mucoadhesiveness, low systemic toxicity, biodegradability, and ability to encapsulate hydrophobic moieties. The neuroprotective efficacy of TQ-PLGA NPs was evaluated in middle cerebral artery occluded rats by pharmacodynamic and biochemical studies together with infarct volume estimation. The pharmacokinetics of TQ-PLGA NPs were determined by analyzing the levels of TQ in brain and blood plasma at various time intervals. The brain localization of PLGA-chitosan nanoparticles (florescent labelled) was determined by confocal microscopy.

## 2. Materials and Methods

### 2.1. Materials

Carboxylic acid terminated PLGA (50 : 50, MW 9–12 KDa) was purchased from Shandong Institute of Medical Instrument (Jinan, Shandong, China). TQ was obtained from Chengdu Kaijie Biopharm Co., Ltd. (Chengdu, China). Polyvinyl alcohol (PVA, MW 30–70 kDa) and low MW CN (~80% degree of deacetylation) were purchased from Sigma Chemical Co. All the other chemicals and reagents used were of the analytical grade.

### 2.2. Animals

Adult male Wistar rats (300–350 g) were selected for the* in vivo* studies. The current study was approved by the Research Review and Ethics Board (RREB), Sun Yat-Sen University, China (approval number: SYSU/2013/203), and all experiments were performed in accordance with the national institutes of health guide for the care and use of laboratory animals.

### 2.3. Preparation of Thymoquinone Loaded PLGA Nanoparticles

TQ-PLGA NPs were prepared by emulsion solvent evaporation method [12]. Briefly, 100 mg of PLGA was dissolved in 1.5 mL of dichloromethane with or without TQ (20% w/w) and added to 15 mL aqueous phase containing 0.25% PVA and 0.25% CN. Since CN alone is unable to stabilize the PLGA nanoparticles, blend of CN and PVA was used for preparation of PLGA-CN nanoparticles [12]. The primary emulsion was vortexed for 90 sec and then sonicated by a probe sonicator at 50 W (Q700 Sonicator, CT USA) for 60 seconds on ice. The organic solvents were evaporated using rotary evaporation (Rotavapor R-124, Buchi, Switzerland) and NPs were freeze-dried using mannitol as cryoprotectant (2.5% w/w).

### 2.4. Characterization of Thymoquinone Loaded PLGA Nanoparticles

TQ-PLGA NPs were characterized for different physiochemical properties such as particle size, size distribution, and zeta potential using Zetasizer (model: Nano ZS, Malvern Instruments, UK). The entrapment efficiency (EE) and loading capacity (LC) were determined by the separation of free TQ from the TQ-PLGA NPs by using a 100 kDa cutoff Millipore membrane filter (Millipore Corp., MA). EE% and LC% were calculated using the following equations:

(1) EE%=total  TQ−free  TQTotal  TQ×100,LC%=total  TQ−free  TQweight  of  TQ-PLGA  NPs×100.

Differential scanning calorimetry (DSC) analysis of PLGA polymer, TQ, physical mixture of polymer + drug (1 : 1), and freeze-dried TQ-PLGA NPs was done by PerkinElmer DSC-7® (PerkinElmer, Inc., MA, USA). Equal weight of each sample was loaded on the standard aluminium pan and crimped. The crimped aluminium pans were heated under continuous nitrogen purging (20 mL/minute) at a heating rate of 5°C/minute to 350°C. An empty crimped aluminium pan served as reference.

The* in vitro* release of TQ from optimized formulation was determined by dialysis bag (MWCO 12 KDa; Sigma-Aldrich) dipped in a dissolution apparatus filled with phosphate buffer (pH 7.4, 37°C ± 0.5°C). At predetermined sampling time points, a 2 mL aliquot was withdrawn for analysis and replaced with equal amount of fresh phosphate buffer till 24 h. The samples were analyzed using reverse-phase HPLC with UV detection at 254 nm using mobile phase water : methanol : 2-propanol (50 : 45 : 5) [13].

### 2.5. Mucoadhesive Potency of Nanoparticles

The mucoadhesive potency of nanoparticles and the TQ solution was determined by their mucin binding efficacy as described by Yin and associates [14]. Briefly, 2 mL of porcine mucin suspension (0.5 mg/mL) in phosphate buffer (pH 7.4) was incubated with same volume of nanoparticle suspension or TQ solution at 37°C for 60 min. After the incubation period, the samples were centrifuged at 60000 ×g for 30 min. The free mucin in the supernatant was measured by UV spectrometry (251 nm) and the mucin binding efficiency was determined by the following equation:

(2) mucin  binding  efficacy%=total  mucin−unassociated  mucintotal  mucin×100.

### 2.6. Pharmacodynamic Study

#### 2.6.1. Animals and Grouping

Rats were subdivided into five groups with 12 rats in each group. Groups 1 and 2 were the sham control and MCAO control, respectively. Group 2 and 3 rats were given TQ-PLGA NPs and TQ solution (equivalent to 5 mg/kg/day TQ) intranasally for 12 days, respectively. Group 4 was given TQ solution orally for 12 days. The dosing of different formulations was started 5 days prior to the MCAO and continued during the 7-day reperfusion time. The intranasal doses (50 *μ*L in each nostril) were given to rats after mildly anaesthetising them under isoflurane. Rats were placed in a supine posture and the doses were instilled with the help of micropipette attached with the polyethylene tube (0.1 mm) positioned 5 mm deep in each nostril [10, 11].

#### 2.6.2. Middle Cerebral Artery Occlusion (MCAO) Surgery

The middle cerebral artery occlusion (MCAO) model was used to evaluate the efficacy of intranasal TQ-PLGA NPs for the neuroprotection and treatment of cerebral ischemia [15]. Amongst many cerebral ischemic stroke models, the intraluminal suture MCAO in rodents, in particular rats, is the most regularly used because it can be used to produce permanent and/or transient ischemia in a very simple manner [15, 16]. Cerebral ischemia in rats was induced by occlusion of middle cerebral artery for 2 h followed by 7 days of reperfusion. Briefly, under an observation microscope, an incision was made in the middle of the neck on ventral side and the external carotid artery, right common carotid artery, and the internal carotid artery were secluded from surrounding tissues. A blunted 4.0 silicon coated nylon monofilament (Doccol Corporation, Pennsylvania Avenue, USA) was inserted into the external carotid artery and slowly pushed towards the middle cerebral artery via the lumen of internal carotid artery until slight resistance was observed, which indicates the occlusion of middle cerebral artery [15]. The rats of sham control underwent all the surgical procedures except the MCAO. The nylon filament was slowly withdrawn after 2 h of induction of ischemia.

#### 2.6.3. Behaviour Activity

After the reperfusion period, the locomotor activity and grip strength were assessed in different groups of animals. For measurement of locomotor activity, each rat was monitored for 5 min by digital photoactometer equipped with infrared light sensitive photocells in a light and sound attenuated room. For measurement of grip strength, the force generated by animal on detaching its front paws from the grid of grip strength meter was recorded and expressed in kilogram unit. After the assessment of neurobehavioral parameters, the animals were sacrificed for determination of ischemia infract volume and biochemical parameters in brain.

#### 2.6.4. Infarct Volume Estimation

The infract volume was measured by a simple staining method described by Bederson et al. [17]. Briefly, rat brain was divided into 2 mm thick slide using microtome and treated with triphenyltetrazolium chloride (TTC, 2% w/v) for 30 min at 37°C. The tissue slides were then fixed with 4% w/v formaldehyde solution. The regions of the slides without the brick red colour (dark colour) of healthy brain tissue were considered infracted and expressed in mm^3^.

### 2.7. Biochemical Estimation

Following the pharmacodynamic tests, the animals were decapitated and the brains were quickly removed, weighed, washed with normal saline, and stored at −80°C till further analysis. Brains were homogenized to a final concentration of 10% w/v using ice cold KCI phosphate buffer (0.1 M pH 7.4). The first supernatant (2000 rpm for 5 min at 4°C) was used for determination of thiobarbituric acid reactive substance (TBARS) and glutathione (GSH) [18, 19]. The second supernatant (11,000 rpm at 4°C for 15 min) was used for estimation of the catalase and super oxide dismutase [18, 19]. The total protein was also determined using the second supernatant by the method described by Lowry et al. [20].

#### 2.7.1. Measurement of Lipid Peroxidation

The TBARS levels in the brain tissue homogenate were determined by the method described by Ohkawa et al. for estimation of lipid peroxidation in the ischemic brain [21]. To 1 mL of brain tissue homogenate, trichloroacetic acid (0.5 mL, 30% w/v) and thiobarbituric acid reagent (0.5 mL, 0.8% w/v) were added. The tubes were kept at a temperature of 80°C for 30 min in a shaking water bath. The tubes were centrifuged at 5000 rpm for 10 min and the amount of TBARS was measured at 540 nm at room temperature. The blank sample contains the entire reagent in the same amount except the brain tissue homogenate. TBARS values were expressed as *n* mol malondialdehyde/gm tissue weight.

#### 2.7.2. Measurement of Reduced Glutathione

The calorimetric method described by Ellman was used for the estimation of GSH in brain tissue homogenate [22]. In the first step, brain homogenate and trichloroacetic acid 10% w/v (1 : 1) were mixed and centrifuged at 5000 rpm for 10 min to separate the proteins. To 100 *μ*L of this supernatant 5,5′-dithiobis nitrobenzoic acid (500 *μ*L), phosphate buffer (pH 7.4, 2 mL), and double distilled water (0.4 mL) were added. The mixture was vortexed and the GSH level was immediately measured at 412 nm against the appropriate blank and expressed as *n* moles × 10^−6^ GSH/gm tissue weight.

#### 2.7.3. Measurement of Catalase

The activity of catalase was determined by a spectrophotometric method described by Greenworld [23]. To 1.9 mL of phosphate buffer (50 mM, pH 7), a 100 *μ*L brain tissue supernatant was added to a disposable plastic cuvette and placed in spectrofluorometer. The reaction was initiated by addition of 1 mL of hydrogen peroxide solution (H_2_O_2_, 30 mM) and the decrease in absorbance of H_2_O_2_ (240 nm) was followed. Catalase values were expressed as *n* moles H_2_O_2_ consumed/min/mg protein.

#### 2.7.4. Measurement of Superoxide Dismutase

The activity of superoxide dismutase was assessed by following the inhibition of pyrogallol autooxidation calorimetrically as described by Kagiyama et al. [24]. A 100 *μ*L brain tissue supernatant was added to 2900 *μ*L of Tris-HCI buffer (pH 8.5). The reaction was started with addition of pyrogallol (25 *μ*L) and the absorbance of pyrogallol was continuously measured at 420 nm for 15 min. The SOD enzyme activity was expressed as U/mg protein.

### 2.8. Brain and Plasma Pharmacokinetics

The brain and plasma pharmacokinetics of TQ-PLGA NPs were determined in rats as described previously [10, 11]. Two separate groups of rats (*n* = 3 for each time point) were given TQ-PLGA NPs (equivalent to 5 mg/kg/day TQ) intravenously (by tail vein) and intranasally, respectively. The pharmacokinetic studies were continued for 72 h to get clear elimination phase for estimation of different pharmacokinetic parameters. Blood (100 *μ*L) was collected by tail vein in precoated heparin tubes and centrifuged for 5 min at 3000 rpm to obtain the plasma (50 *μ*L) for estimation of TQ. For brain collection, rats were sacrificed at scheduled time intervals by cervical dislocation. Brain was dissected out from skull, washed, and homogenised with 2 mL of normal saline by a tissue homogeniser. A small aliquot of brain tissue homogenate (200 *μ*L) was used for estimation of TQ levels in brain. TQ was analyzed in plasma and brain by reverse-phase HPLC with UV detection at 254 nm [13]. The peak plasma concentration (*C*_max_) and the time required to reach the peak plasma (*T*_max_) were obtained from the brain/plasma concentration-time profile. The drug targeting efficacy (DTE%) and nose to brain direct transport percentage (DTP%) of the TQ-PLGA NPs after intranasal administration were calculated as follows [10]:

(3) drug  targeting  efficiencyDTE%=AUC  brain/AUC  plasmai.n.AUC  brain/AUC  plasmai.v.×100,direct  transport  percentageDTP%=Di.n.−DzDi.n.×100,

where *D*_*z*_ = (*D*_i.v._/*P*_i.v._) × *P*_i.n._ and *D*_*z*_ is the brain AUC fraction contributed by systemic circulation through the BBB following intranasal administration. *D*_i.v._, *P*_i.v._, *D*_i.n._, and *P*_i.n._ correspond to the AUC_0–72 h_ (brain/i.v.), AUC_0–72 h_ (plasma/i.v.), AUC_0–72 h_ (brain/i.n.), and AUC_0–72 h_ (plasma/i.n.).

### 2.9. Qualitative Localization by Confocal Laser Microscopy

The qualitative localization of florescent labelled PLGA NPs was determined in brain tissues by confocal laser microscopy (Olympus Fluoview™, Melville, NY, USA) [25, 26]. For preparation of DAPI (4-6-diamidino-2-phenylindole) loaded PLGA nanoparticles, 0.2 mL of DAPI solution (50 *μ*g/mL in ethanol) was dissolved in PLGA solution and nanoparticles were prepared in similar way as described earlier. For this study, cohort of rats (normal non-MCAO rats, *n* = 2) were given DAPI loaded PLGA NPs by intravenous and intranasal route and sacrificed at two time points, that is, after 60 min and 4 hr, respectively. The brain was extracted and immediately stored at −80°C to prevent loss of fluorescence. On the day of experiment, coronal section (5 mm) of brain was made with a cryostat and analyzed with fluorescence microscope.

### 2.10. Statistical Analysis

All the* in vivo* data are expressed as mean ± SEM. One-way analysis of variance (ANOVA) and Dunnett's “*t*”-test were used for the comparison of different experimental groups. *P* < 0.05 was considered to represent significant difference, whereas *P* < 0.01 represents a highly significant difference between groups, respectively.

## 3. Results

### 3.1. Optimized Thymoquinone Loaded PLGA Nanoparticles

For preparation of TQ-PLGA nanoparticles, at first the blank, PLGA nanoparticles were synthesized and concentrations of polymer and stabiliser were optimized. The optimized blank nanoparticles have PLGA : CN ratio of 1 : 1 (CN : PVA = 1 : 1) with particles size of 154.3 ± 6.5 nm and zeta potential of +38.56 ± 3.32 mV. In the next step, TQ loaded PLGA nanoparticles were prepared by dissolving TQ in polymer phase (drug : polymer 0.3 : 1) and using the same condition/concentration as optimized for placebo nanoparticles (Table 1). The mean particles size of TQ-PLGA NPs (183.5 ± 8.2) was found to be larger than the blank nanoparticles due to increase in the concentration of internal phase. The average zeta potential values were also found to be lower than the placebo nanoparticles due to TQ mediated increase in the net negative charge of the emulsion. Nevertheless, zeta potential value of 33.63 ± 2.25 mV (Table 1) represents a stable system. The PDI value of 0.257 represents narrow and unimodel particle size distribution for minimal deviation in pharmacokinetic parameters between different rats. EE% and LC% of the optimized TQ-PLGA NPs were found to be 73.2 ± 2.6 and 31.4 ± 2.1, respectively, which shows that TQ can be entrapped within the PLGA-CN nanoparticles. The mucin binding efficacy for optimized TQ-PLGA NPs, PLGA NPs, and TQ solution was found to be 59.6 ± 1.5%, 64.0 ± 2.7%, and 2.3 ± 0.6%, respectively. Thus, the mucoadhesive strength of TQ solution was inadequate to provide sufficient nasal retention time for prolonged therapeutic effect. TQ-PLGA NPs had lower mucoadhesive strength than PLGA NPs due to reduction in their net positive charge.

**Figure 1:**
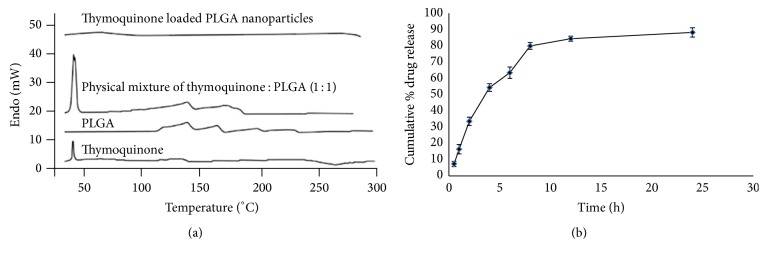
(a) DSC thermogram of TQ, PLGA, and physical mixture of TQ-PLGA and TQ loaded PLGA nanoparticles, respectively. TQ was molecularly distributed with the PLGA polymer and entrapped within PLGA-CN nanoparticle matrix; therefore the characteristic peaks of TQ (45–47°C) and PLGA (~120°C) were not seen in TQ-PLGA nanoparticles. (b) The* in vitro* release of TQ-PLGA nanoparticles. It showed a biphasic release with 16% TQ release within 1 h followed by characteristic sustained release for more than 24 h.

Figure 1(a) shows the DSC thermograms of TQ, PLGA, TQ : PLGA mixture, and TQ-PLGA NPs. The melting point of pure TQ is 45–47°C and thus the endothermic peak at ~45°C corresponds to pure TQ. PLGA polymer showed endothermic peak at ~120°C, whereas the physical mixture of TQ + PLGA retained the characteristic peaks of both TQ and PLGA. Conversely, TQ was molecularly distributed with the PLGA polymer and entrapped within PLGA-CN nanoparticle matrix; therefore the characteristic peaks of TQ and PLGA were not seen in TQ-PLGA NPs. The* in vitro* release of TQ from optimized TQ-PLGA NPs (Figure 1(b)) showed a biphasic release pattern with 16% drug release within 1 h followed by characteristic sustained release for more than 24 h. The cumulative percentage release of TQ from TQ-PLGA NPs was 88.21 ± 2.872% over a period of 24 h. The initial burst drug release was most likely due to the release of TQ loosely attached to the surface of the nanoparticles, whereas the later slow release may be due to the TQ release from the core of PLGA nanoparticles by the swelling and hydration of PLGA NPs matrix. These results showed that TQ was effectively entrapped within the PLGA NPs matrix.

### 3.2. Pharmacodynamic Study and Infract Volume Measurement

**Figure 2:**
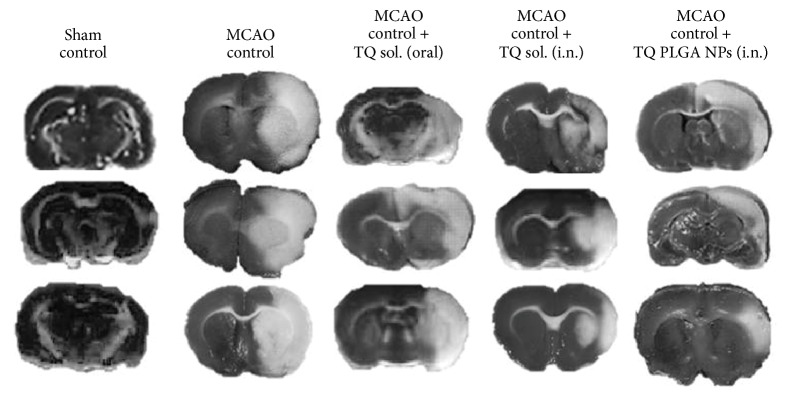
The representative images of TTC staining of different groups. The regions of the slides with the white colour are considered infracted and those with dark colour represent the healthy brain tissues. The intranasal delivery of TQ-PLGA nanoparticles led to significant reduction in infract volume in the MCAO rats when compared to TQ solution delivered by intranasal or oral route alone.

A significant reduction in the locomotor count (*P* < 0.01) and grip strength (*P* < 0.05) was observed in the MCAO rats in comparison to the sham rats (Table 2). The difference between the locomotor count and grip strength between sham group and MCAO rats showed that ischemia symptoms were evident only after middle cerebral artery occlusion. Pretreatment with TQ-PLGA NPs (i.n.) led to significant improvement in locomotor counts and grip strength in comparison to the MCAO rats (Table 2). TQ-PLGA NPs (oral) also improved the locomotor count and grip strength but were less effective than TQ-PLGA NPs (i.n.). Similarly, TTC staining also showed a widespread infract area in MCAO rats (~76 mm^3^) seven days after reperfusion (Figure 2 and Table 2). The treatment of MCAO rats with TQ-PLGA NPs (i.n.), TQ solutions (i.n.), and TQ solutions (oral) reduced the infract volume by 49.7% (*P* < 0.05), 40.7%, and 41.2%, respectively.

### 3.3. Biochemical Studies

**Figure 3:**
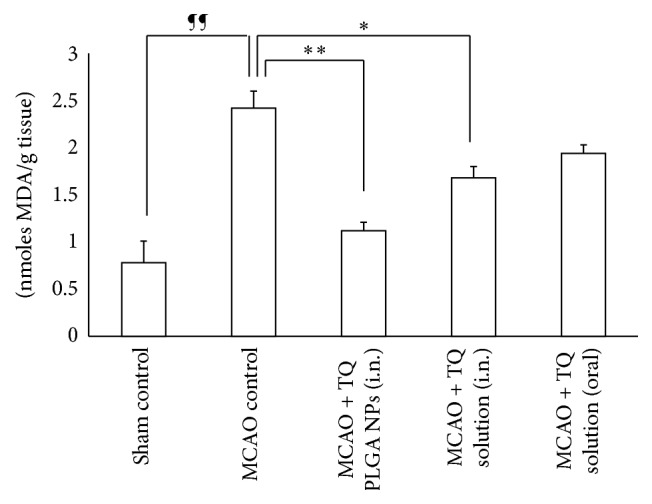
Effect of TQ loaded PLGA nanoparticles on TBARS level in the brain of MCAO rats. The intranasal delivery of TQ-PLGA nanoparticles led to significant reduction in TBARS levels in the MCAO rats when compared to TQ solution delivered by intranasal or oral route alone (*N* = 6; data represent mean ± SEM; ^¶¶^ *P* < 0.01 versus sham control; ^*∗∗*^ *P* < 0.01 versus MCAO control; and ^*∗*^ *P* < 0.05 versus MCAO control).

The brain lipid peroxidation was assessed by measurement of TBARS levels in all the groups 7 days after MCAO. The TBARS levels were found to be 200% higher (*P* < 0.01) in the MCAO rats in comparison to the sham rats (Figure 3). Pretreatment with TQ-PLGA NPs (i.n.) led to significant reduction in TBARS levels in the MCAO rats (Figure 2). TQ solution delivered by oral route also reduced the TBARS formation in MCAO rats but was less effective than TQ-PLGA NPs (i.n.).

**Figure 4:**
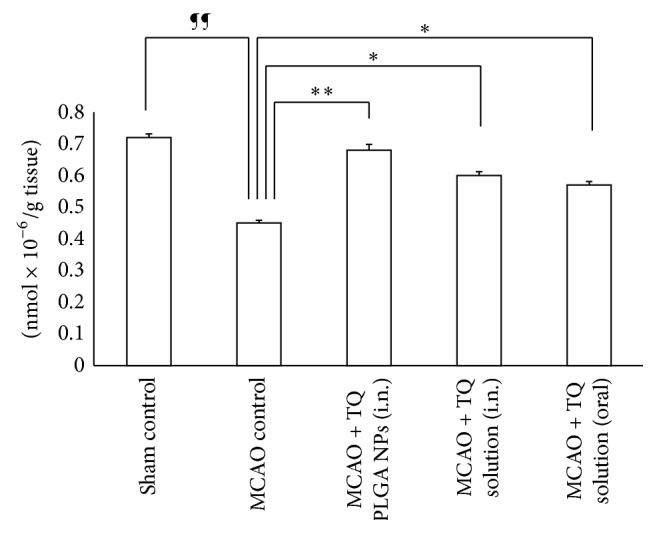
Effect of TQ loaded PLGA nanoparticles on glutathione level in the brain of MCAO rats. The intranasal delivery of TQ-PLGA nanoparticles led to significant elevation in the GSH levels in the MCAO rats when compared to TQ solution delivered by intranasal or oral route alone (*N* = 6; data represent mean ± SEM; ^¶¶^ *P* < 0.01 versus sham control; ^*∗∗*^ *P* < 0.01 versus MCAO control; and ^*∗*^ *P* < 0.05 versus MCAO control).

GSH in the brain of MCAO rats was 42% lower than the sham control rats (*P* < 0.01). A number of publications have shown that overproduction of ROS is counter-balanced by endogenous antioxidants causing their depletion from the cells [27]. In this case endogenous GSH was consumed due to scavenging of rapidly generating free radical species in cerebral infarct. Intranasal delivery of TQ-PLGA NPs significantly elevated the glutathione levels in MCAO rats (*P* < 0.01) (Figure 4). TQ solution delivered by nasal or oral route was also effective in enhancing the levels of glutathione in the MCAO rats.

The SOD and catalase activity of different groups of rats are provided in Table 3. The catalase and SOD enzyme activity were significantly decreased in the MCAO rats in contrast to the sham control rats. Treatment with TQ-PLGA NPs significantly elevated the activity of both enzymes in the MCAO rats.

### 3.4. Brain and Plasma Pharmacokinetics

**Figure 5:**
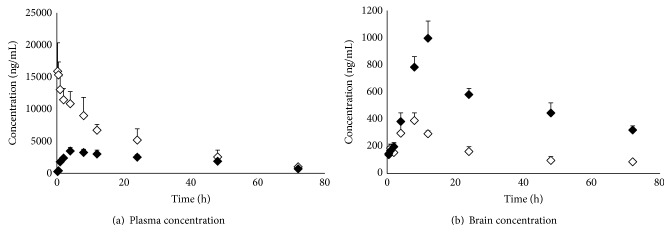
Brain and plasma levels of TQ following the administration of TQ loaded PLGA nanoparticles by (a) intravenous route (white diamonds) and (b) intranasal route (black diamonds). The intranasal delivery of TQ-PLGA nanoparticles led to higher brain concentration but lower plasma concentration of TQ when compared with intravenous delivery (*N* = 3; data represent mean ± SEM).

The concentration of TQ in brain and blood plasma was determined at different time points till 72 h after dosing the TQ-PLGA NPs by intranasal and intravenous route, respectively (Figure 5(b)). A high brain TQ concentration was obtained when TQ-PLGA NPs were delivered by intranasal route (Figure 5(b)). Other pharmacokinetic parameters for TQ, for example, half-life, AUC, and AUMC, were found to be higher in brain for TQ-PLGA NPs (i.n.) in comparison to intravenous delivery (Figure 5(a)). Also, the rate of elimination of TQ-PLGA NPs (i.n.) was found to be significantly lower in brain (Table 4). Based on plasma AUC values, the systemic bioavailability of TQ-PLGA NPs (i.n.) was found to be 28.07%. The DTE shows the average partitioning of TQ between brain and blood with time, whereas DTP stands for the percentage of TQ directly carried to the brain by nose to brain pathways. The DTE and DTP for TQ-PLGA NPs (i.n.) were calculated to be 524.17 and 80.47, respectively.

### 3.5. Qualitative Localization in Brain

**Figure 6:**
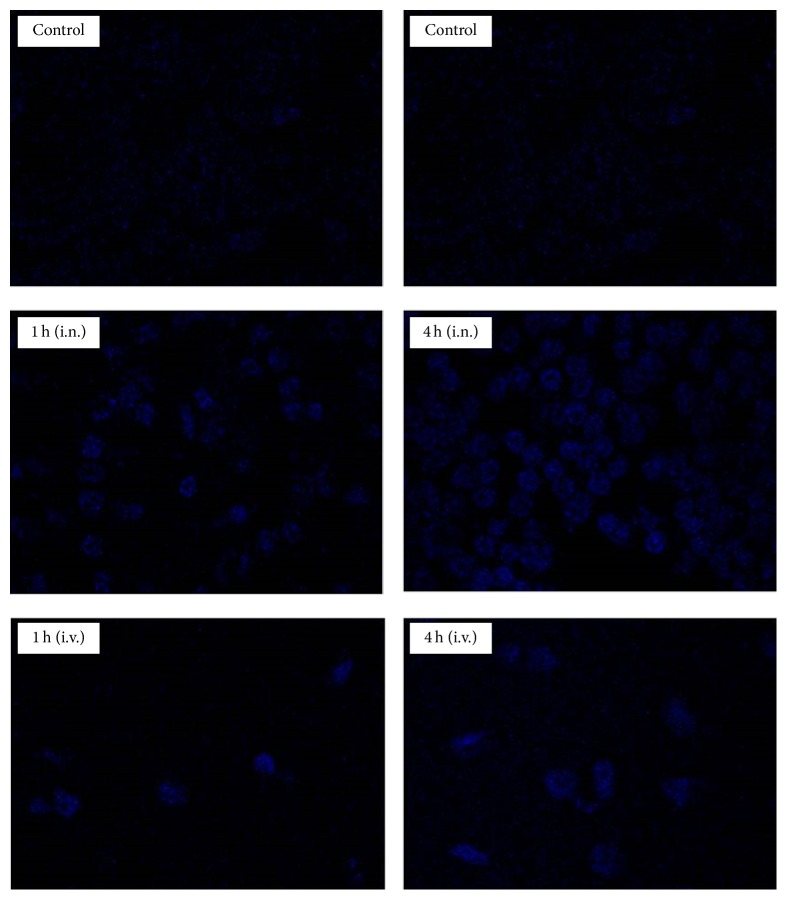
Qualitative localization of DAPI loaded PLGA nanoparticles in brain tissues after intranasal (i.n.) and intravenous (i.v.) administration at 1 h and 4 h, respectively, after dosing. A higher proportion of DAPI stained brain cells were seen when DAPI loaded PLGA nanoparticles were administered by i.n. route in comparison to i.v. route.

The intranasal administration of DAPI-PLGA NPs showed intense blue florescence in brain due to significant role of olfactory and trigeminal pathways in nose to brain transportation of DAPI-PLGA NPs (Figure 6) [9], although the intensity of blue florescence was higher at 4 h than 1 h after dosing of DAPI-PLGA NPs (i.n.). In comparison, a low intensity of blue florescence was seen in the brain when rats were treated with DAPI loaded PLGA NPs (i.v.) due to the restriction imposed by BBB.

## 4. Discussion

The cerebral ischemia and reperfusion have been reported to elevate the free radical production in brain tissues and lead to substantial alterations in the level as well as activity of endogenous antioxidant enzymes [2, 3], whereas in normal brain tissues free radical species and ROS are neutralized by endogenous enzymatic and nonenzymatic antioxidants [2, 3]. ROS species, for example, superoxide, hydroxyl, and peroxynitrite radical, have established role in peroxidation of cell membrane lipids [2, 3, 27]. Oxidative stress by brain ischemia has been found to encourage the synthesis of peroxynitrite, a powerful free radical species capable of inducing severe lipid peroxidation and cellular damage to the brain [28]. Brain tissues are more prone towards oxidative injury due to their high polyunsaturated fatty acids content and low repair mechanism together with nonreplicating nature of neuron [4]. TQ is a hydrophobic phytochemical and many natural hydrophobic phytochemicals delivered by oral route have been found to have low plasma bioavailability and poor permeability across intestinal tract [29]. In a recent report by Alkharfy and colleagues, TQ was reported to have very slow absorption but rapid elimination following oral delivery [30]. They further reported the absolute bioavailability of TQ to be <60% of the administered dose with >99% plasma protein binding [30]. Therefore, intranasal nose to brain route represents a better strategy for noninvasive delivery of TQ to the brain. The literature suggests that an intranasally administered therapeutic agent reaches the brain by a number of extracellular and intracellular mechanisms by olfactory and trigeminal nerve pathways [10, 11]. A number of studies have comprehensively shown that mucoadhesive nanoparticles can transport the enclosed drugs to the brain by unique nose to brain transportation pathways and maintain effective therapeutic concentration beyond 48 h in the brain [9–11]. Therefore, TQ was encapsulated in polymeric nanoparticles composed of PLGA and CN. Nanoparticulate drug delivery systems have been documented to have higher potential in treatment of ischemic stroke and, potentially, other neurologic disorders. For instance, Joachim et al. showed that gelatin nanoparticle improved the neuroprotection of intranasally delivered osteopontin in rat ischemic stroke model [31]. In a recent study, it was shown that intranasal delivery of PNIPAM nanoformulation of curcuminoids reduced oxidative stress-associated brain injury after middle cerebral artery occlusion [32]. Zhao et al. reported that intranasal delivery of gelatin nanoparticle loaded with neuropeptide substance P enhanced the neurorecovery in hemiparkinsonian rats [33]. Nagai et al. showed that intravenous administration of cilostazol nanoparticles ameliorates acute ischemic stroke in a cerebral ischemia/reperfusion-induced injury model [34]. Consistent with these findings, pretreatment with TQ-PLGA NPs (i.n.) improved the locomotor activity and grip strength in the MCAO rats with reversal of ischemic infarct in the brain. TQ and its active metabolite thymohydroquinone have been found to inhibit the lipid peroxidation as they scavenge the superoxide, hydroxyl radical, and singlet molecular oxygen [5, 6]. Beside this, TQ has also been reported to restrain the arachidonic acid metabolism and prevent the ischemic brain damage from metabolic products formed by inflammatory cyclooxygenase and lipoxyegnase pathways [35]. Therefore, pretreatment with TQ-PLGA NPs led to reversal in the TBARS and GSH level in the MCAO rats. The potency of the reversal in the GSH and TBARS level was strongly dependent on the uninterrupted availability of TQ at the site of ischemia infract, which explains the superior protective and therapeutic effect of TQ-PLGA NPs (i.n.) in comparison to other formulations [5, 18]. These observations are in harmony with the previous publications which have comprehensively shown that treatment with TQ rescued the neurons from ischemic death and offered protection against cerebral ischemia [5, 6, 18, 19]. Although, like previous studies, we also observed improvement in the pharmacodynamic and biochemical profile after oral administration of TQ, the results were inferior in comparison to TQ-PLGA NPs (i.n.) [5, 18, 19]. Mucociliary clearance as well as low mucoadhesive strength of the TQ solution led to its rapid clearance from the nasal cavity and diminished therapeutic activity [10, 11]. The pharmacokinetic studies showed that the intranasal administration of TQ-PLGA NPs facilitated the delivery of TQ to brain and increased its brain concentration for a prolonged period of time with simultaneous improvement in other pharmacokinetic parameters in brain. The high mucoadhesive strength of PLGA-CN nanoparticles increased the retention time of TQ in the nasal cavity to sustain effective therapeutic concentration in brain. In addition, CN used in nose to brain formulations have been found to enhance the paracellular transport through epithelial tight junctions due to their specific interaction with the protein kinase C pathway or electrostatic interaction with negative charged sialic acid residues on mucosal epithelial cells [36]. Several studies have shown that NPs smaller than 200 nm are easily transported through olfactory membrane transcellularly by olfactory neurones to the brain and the results of qualitative localization study in brain tissues with DAPI loaded PLGA nanoparticles (i.n.) are consistent with these findings [9–11]. Thus, PLGA-CN nanoparticles improved the delivery of TQ to brain which potentiated its neuroprotective and therapeutic effect against cerebral ischemia.

## 5. Conclusion

Cerebral ischemia is one the most challenging CNS diseases as there is a large clinical variability in terms of severity of ischemia, localization, and duration with limited treatment options. In the current study, nose to brain delivery of optimized thymoquinone loaded PLGA nanoparticles enhanced the pharmacokinetic profile of thymoquinone in brain and effectively reversed the symptoms of cerebral ischemia as well as rescuing the brain cells from ischemic death in rats by virtue of their antioxidant and free radical scavenging properties. Thus, antioxidant therapy with novel nanomedicine and new route of administration (i.e., nose to brain delivery) can be potentially used for neuroprotection and treatment of cerebral ischemia. However, these novel nanomedicines require rigorous preclinical studies for their toxicity, pharmacokinetics, and pharmacodynamics in higher animal models for possible human clinical application.

## Figures and Tables

**Table 1:** Concentration of ingredients and physiochemical parameter of the optimized thymoquinone loaded PLGA nanoparticles.

Conc. of polymers	Conc. of stabiliser	Conc. of drug (mg/mL)	Mean particle size (nm ± SD)	Mean PDI (±SD)	Zeta potential (mV ± SD)	EE% (±SD)	LC% (±SD)
1 mg/mL	0.25 mg/mL (CN) 0.25 mg/mL (PVA)	0.3 mg/mL	183.5 ± 8.2	0.257 ± 0.02	33.63 ± 2.25	73.2 ± 2.6	31.4 ± 2.1

The optimized TQ-PLGA nanoparticles had PLGA : stabiliser : TQ ratio of 1 : 0.5 : 0.3 with mean particle size of 183.5 nm, polydispersity index (PDI) of 0.25, and zeta potential of +33.6 mV. The average encapsulation efficiency and loading capacity of TQ-PLGA nanoparticles were found to be 73.2% and 31.4%, respectively.

**Table 2:** Effect of thymoquinone loaded PLGA nanoparticles on locomotor activity, grip strength, and cerebral infract volume in the MCAO rats.

Groups	Treatment	Locomotor activity (count/5 min)	Grip strength (Kg/unit)	Infarct volume (mm^3^)^#^
1	Sham control	21.62 ± 0.312	0.68 ± 0.053	—
2	MCAO control	3.54 ± 0.021^¶¶^	0.12 ± 0.03^¶¶^	76.28 ± 0.112
3	MCAO + TQ PLGA NPs (i.n.)	16.89 ± 0.218^*∗∗*^	0.36 ± 0.087^*∗*^	38.34 ± 0.123^*∗*^
4	MCAO + TQ solution (i.n.)	15.12 ± 0.093^*∗*^	0.19 ± 0.101	45.19 ± 0.091
5	MCAO + TQ solution (oral)	9.23 ± 0.101^*∗*^	0.23 ± 0.214^*∗*^	44.78 ± 0.153

The intranasal delivery of TQ-PLGA nanoparticles led to significant increase in the locomotor activity and grip strength and decrease in ischemia infarct volume in the MCAO rats when compared to TQ solution delivered by intranasal or oral route alone. *N* = 12 (^#^ *N* = 6); data represent mean ± SEM; ^¶¶^ *P* < 0.01 versus sham control;   ^*∗∗*^ *P* < 0.01 versus MCAO control; and  ^*∗*^ *P* < 0.05 versus MCAO control.

**Table 3:** Effect of thymoquinone loaded PLGA nanoparticles on the catalase and SOD activity in the brain of MCAO rats.

Groups	Treatment	Catalase (*n* moles of H_2_O_2_/min/mg protein)	SOD (U/mg protein)
1	Sham control	3.81 ± 0.112	7.30 ± 0.031
2	MCAO control	1.54 ± 0.106^¶¶^	4.53 ± 0.311^¶¶^
3	MCAO + TQ PLGA NPs (i.n.)	3.64 ± 0.054^*∗*^	7.01 ± 0.078^*∗∗*^
4	MCAO + TQ solution (i.n.)	2.06 ± 0.018	5.57 ± 0.031^*∗*^
5	MCAO + TQ solution (oral)	1.96 ± 0.021	5.49 ± 0.084^*∗*^

The intranasal delivery of TQ-PLGA nanoparticles led to significant increase in the antioxidant enzymes catalase and SOD in the MCAO rats when compared to TQ solution delivered by intranasal or oral route alone. *N* = 6; data represent mean ± SEM; ^¶¶^ *P* < 0.01 versus sham control;  ^*∗∗*^ *P* < 0.01 versus MCAO control; and  ^*∗*^ *P* < 0.05 versus MCAO control.

**Table 4:** Brain and plasma pharmacokinetic parameters of thymoquinone loaded PLGA nanoparticles after intravenous and intranasal delivery.

Formulation	Organ/tissue	Peak plasma conc. (*C*_max_) (ng/mL)	Time peak plasma conc. (*T*_max_) (h)	Half-life (*T*_1/2_) (h)	Rate of elimination (*K*_*e*_) (h^−1^)	AUC_0–72 h_ (ng mL^−1^ h)	AUC_0–*∞*_ (ng mL^−1^ h)
TQ-PLGA NPs (i.v.)	Brain	390.61 ± 54.44	8	18.11 ± 1.97	0.010 ± 0.003	11204.3 ± 3211.6	20296.3 ± 3265.6
	Plasma	15897.67 ± 3443.52	—	62.34 ± 7.04	0.038 ± 0.004	312654.2 ± 6542.6	351453.2 ± 14915.1

TQ-PLGA NPs (i.n.)	Brain	996.43 ± 119.36	12	118.23 ± 3.97	0.005 ± 0.000	33771.2 ± 994.5	98675.6 ± 8157.1
	Plasma	3465.33 ± 664.16	4	194.24 ± 9.87	0.003 ± 0.000	142315.1 ± 33217.3	359773.4 ± 44132.3

*N* = 3; data represent mean ± SEM.
